# Inbreeding rate modifies the dynamics of genetic load in small populations

**DOI:** 10.1002/ece3.293

**Published:** 2012-07-01

**Authors:** Nina Pekkala, K Emily Knott, Janne S Kotiaho, Mikael Puurtinen

**Affiliations:** 1 Department of Biological and Environmental Science, University of JyväskyläFinland; 2 Natural History Museum, University of JyväskyläFinland; 3 Centre of Excellence in Biological Interactions, University of JyväskyläFinland

**Keywords:** *Drosophila littoralis*, extinction, genetic drift, inbreeding depression, offspring production, purging

## Abstract

The negative fitness consequences of close inbreeding are widely recognized, but predicting the long-term effects of inbreeding and genetic drift due to limited population size is not straightforward. As the frequency and homozygosity of recessive deleterious alleles increase, selection can remove (purge) them from a population, reducing the genetic load. At the same time, small population size relaxes selection against mildly harmful mutations, which may lead to accumulation of genetic load. The efficiency of purging and the accumulation of mutations both depend on the rate of inbreeding (i.e., population size) and on the nature of mutations. We studied how increasing levels of inbreeding affect offspring production and extinction in experimental *Drosophila littoralis* populations replicated in two sizes, *N* = 10 and *N* = 40. Offspring production and extinction were measured over 25 generations concurrently with a large control population. In the *N* = 10 populations, offspring production decreased strongly at low levels of inbreeding, then recovered only to show a consistent subsequent decline, suggesting early expression and purging of recessive highly deleterious alleles and subsequent accumulation of mildly harmful mutations. In the *N* = 40 populations, offspring production declined only after inbreeding reached higher levels, suggesting that inbreeding and genetic drift pose a smaller threat to population fitness when inbreeding is slow. Our results suggest that highly deleterious alleles can be purged in small populations already at low levels of inbreeding, but that purging does not protect the small populations from eventual genetic deterioration and extinction.

## Introduction

An increasing number of plant and animal populations are decreasing in size and becoming isolated from each other, mostly due to anthropogenic destruction and fragmentation of natural habitats (Ewers and Didham 2006). Small and isolated populations face an elevated risk of extinction due to both demographic and genetic reasons (Lande 1988; Hedrick 1994; Newman and Pilson 1997; Saccheri et al. 1998; Bijlsma et al. 2000; Amos and Balmford 2001; Spielman et al. 2004; Frankham 2005; O'Grady et al. 2006). Two important genetic mechanisms acting on short to intermediate timescales and threatening the persistence of small populations are inbreeding depression and increased genetic load due to genetic drift. Inbreeding depression, the reduced fitness of offspring from mating between close relatives, is in general mainly caused by the unmasking of recessive deleterious alleles in homozygous genotypes, although loss of heterozygosity in overdominant loci and negative epistatic interactions between homozygous loci may also be involved (Charlesworth and Willis 2009). The level of in breeding inevitably increases in small populations even with random mating, as only a limited number of individuals contribute to each generation. Genetic load in small populations is increased as a consequence of relaxed natural selection leading to the accumulation and fixation of harmful mutations (Whitlock 2000).

Although inbreeding depression and accumulation of deleterious mutations by genetic drift are predicted to decrease the fitness of small populations, small population size can also enhance selection against recessive and partially recessive deleterious alleles as they are expressed in homozygous condition (Hedrick 1994, 2002; Wang et al. 1999; Kirkpatrick and Jarne 2000; Glemin 2003). The purging of deleterious alleles from a population can thus counteract the negative effects of inbreeding and genetic drift. The contrasting effects of inbreeding depression, accumulation of deleterious alleles, and purging mean that predicting the long-term effects of reduced population size on fitness and viability of populations is not straightforward.

Theoretical models and simulations predict that inbreeding rate (i.e., the size of the population) is one of the key factors affecting purging and accumulation of mutations. In general, slow inbreeding (i.e., large population size) is predicted to be less detrimental than fast inbreeding (i.e., small population size) when the populations are compared at the same inbreeding coefficient (Wang et al. 1999; Theodorou and Couvet 2006). This is because slow inbreeding allows more time for selection to purge the deleterious alleles from the populations, and because in these populations a larger proportion of mutations are under effective selection due to their larger effective size (Wang et al. 1999).

In addition to inbreeding rate, the harmfulness of mutations is predicted to have significant effects on fitness of small populations. Highly deleterious recessive mutations can depress fitness substantially during the first generations of reduced population size, but they are also efficiently purged, and hence pose little threat to the long-term survival of populations, provided that the populations survive past the initial purging stage (Wang et al. 1999; Theodorou and Couvet 2006). Mildly deleterious mutations, on the other hand, are not efficiently purged and are predicted to cause a continuous decrease in fitness, presenting a serious threat to the survival of small populations (Lande 1994). The most damaging mutations for population viability are those that are just mild enough for their frequency to be dominated by genetic drift (Kimura et al. 1963; Lande 1994, 1998).

Theoretical predictions about long-term viable population sizes are highly sensitive to the mutation parameters assumed (Hedrick 1994; Lande 1994, 1998; Lynch et al. 1995a,b; Wang et al. 1999; Bataillon and Kirkpatrick 2000; Whitlock 2000; Glemin 2003; Theodorou and Couvet 2006). Unfortunately, we have a very vague idea about the relevant mutation parameters in real populations (Eyre-Walker and Keightley 2007), and thus cannot rely on predictions of theoretical models in determining the long-term viability of populations with reduced population size. Therefore, long-term experiments over different population sizes have been called for to validate the basic predictions of the theoretical models (Lynch et al. 1995a, p. 513).

Current empirical evidence suggests that the effectiveness of purging in small populations is relatively unpredictable (reviewed in Ballou 1997; Byers and Waller 1999; Crnokrak and Barrett 2002; Leberg and Firmin 2008). In their review of experimental studies, Crnokrak and Barrett (2002) conclude that purging probably does happen in small populations, but in which situations and to what extent purging is likely to occur remain open questions. Experimental studies on the effects of inbreeding rate on fitness and population viability have also provided conflicting results. In some studies slower inbreeding has been less harmful to the fitness of individuals or viability of populations (Ehiobu et al. 1989; Day et al. 2003; Reed et al. 2003; Pedersen et al. 2005), whereas in others, the effects of inbreeding rate have been highly variable (negative, positive, or nonsignificant) depending on the trait and the environment (Bijlsma et al. 2000; Swindell and Bouzat 2006c; Mikkelsen et al. 2010; Kristensen et al. 2011). Most of the previous studies have measured the fitness of individual populations only at one point in time, at a given coefficient of inbreeding (for notable exceptions see Bryant et al. 1999; Reed et al. 2003; Larsen et al. 2011). In contrast to such point estimates, monitoring the evolution of population fitness from low to increasing levels of inbreeding may provide a better understanding of the dynamics of genetic load in small populations. In addition, we have little knowledge about the effects of very low levels of inbreeding, as most studies have focused on relatively high inbreeding coefficients (most often *f* ≥ 0.25; but see, e.g., Bijlsma et al. 2000; Larsen et al. 2011)

We studied the effect of population size on the relationship between inbreeding level and population viability in experimental *Drosophila littoralis* populations. Population viability, measured as offspring production and extinction, was followed in populations replicated in two sizes (*N* = 10 and *N* = 40) for 25 generations concurrently with a large control population. The effect of population size on population viability was assessed over a range (*f* = 0.06–0.42) of inbreeding coefficients estimated from genetic variation at microsatellite loci. In the smaller populations, the estimated inbreeding coefficient reached a value of 0.78 by generation 25.

## Materials and Methods

### Population size treatments

A laboratory population of the boreal drosophilid *D. littoralis* was founded in spring 2006 from 157 males and 99 females collected from a natural population by the Tourujoki River in Jyväskylä, Central Finland. The flies were maintained in laboratory at 19°C and relative humidity of 60%, with constant light and malt medium (Lakovaara 1969) available ad libitum. Thirty-four of the 99 females had been inseminated in the wild and produced fertile eggs after transfer to the laboratory. The rest of the females were mated randomly with the wild-caught males. For the first five generations (P-F4), the population was maintained in a pedigree. Inbreeding was reduced by excluding matings between full siblings. Population size was increased to 419 breeding pairs in F2, and maintained as 396 pairs in F3 and 368 pairs in F4. For the next two generations (F5–F6), the flies were allowed to mate randomly (with separate generations) as a population of approximately 500 breeding pairs.

At the seventh laboratory generation (F7, from here on referred to as generation 0), we established different-sized experimental populations from the large population: 16 replicate populations of five pairs each (N10) and 12 replicate populations of 20 pairs each (N40). A large population consisting of 250 pairs (N500) was also established to serve as a control for possible temporal environmental effects (Lynch and Walsh 1998, p. 263; Crnokrak and Barrett 2002). To minimize the increase in the level of inbreeding in the control population, we decided to have only one control population and maintain it as large as possible, rather than to have multiple smaller control populations. As the control population was distributed into 50 bottles (see below), the stochastic, nonenvironmental variation in offspring production is expected to be minor relative to unintentional environmental variation that is always present even in highly controlled laboratory conditions (e.g., minor fluctuations in the quality of the food medium).

### Population maintenance

The populations were maintained with separate generations for a total of 26 generations (generations 0–25), keeping the population size constant according to the population size treatment. Each new generation was started with randomly chosen flies from the previous generation. The populations were maintained in plastic bottles containing 50 mL of malt medium (Lakovaara 1969). All populations were maintained at the same density of five pairs per bottle. Thus, the N10 populations consisted of one bottle per replicate, the N40 populations consisted of four bottles per replicate, and the control population consisted of 50 bottles. In addition, one to three extra bottles were set up for each N10 replicate population at each generation (when enough adult flies were available) to get flies for other experiments, and to protect against possible handling accidents during population maintenance. However, all the bottles (including the extra bottles) of each N10 population were always formed from flies collected from a single randomly chosen bottle that was designated a priori to serve as the source of flies for the next generation. Only in the case this bottle did not produce at least five males and five females (enough flies to start the next generation), a replacement bottle was randomly drawn from among the extra bottles. The N40 and the control (N500) populations were kept panmictic by mixing the offspring from all bottles in the same replicate prior to collecting the flies for the subsequent generation.

For the first 10 generations (generations 0–9), we maintained the populations as follows: At the start of each generation, five mature (age 16–23 days from eclosion), randomly chosen males and females from the previous generation were introduced to the bottle to mate and lay eggs. After 5 days in the bottle, the parental flies were removed. Under the rearing conditions used, the egg-to-adult development time of the flies was approximately 3 to 4 weeks. To avoid selection for early reproduction and fast egg-to-adult development, and to time our collection of the offspring to the peak emergence time, the first eclosed offspring from each population were counted and discarded 21 days after the removal of parental flies. Seven days later (28 days since the removal of parental flies), all newly eclosed offspring were collected, counted, and separated according to sex under CO_2_ anesthesia. Based on a preliminary experiment (results not shown), under the rearing conditions used, *D. littoralis* males mature at the earliest 10 days after eclosion. Thus, as the offspring were collected 0–7 days after eclosion, they were assumed to be virgins. The collected flies were kept in plastic vials (diameter 23.5 mm, height 75.0 mm, 8 mL of malt medium) at maximum density of 10 flies per vial, and changed to fresh vials every 7 days. Sixteen days from collection, the offspring flies were used to start the next generation, keeping the breeding population size constant according to the population size treatment.

Ten generations from establishment of the experimental populations, we noticed that the peak emergence time of the flies had advanced. We implemented no direct selection on timing of reproduction or on egg-to-adult development time of the flies. However, the random collection of parental flies for each generation may have caused positive selection on fecundity of the flies. If a genetic correlation exists between fecundity and development time, unintentional selection on fecundity may have caused correlated evolution of faster development. To maintain the collection of flies at the peak emergence time (and to avoid causing a false increase in population extinction risk), we changed the procedure from generation 10 onwards, so that we collected offspring that eclosed between 17 and 24 days after removal of the parental flies, that is, 4 days earlier than before. We kept the generation length constant by starting the next generation 20 days from the collection of the flies. Thus, the age of the flies at introduction to the bottle was now 20–27 days from eclosion. The difference between the two procedures is minor, as the age of the flies at collecting is the same (0–7 days from eclosion), and the age at introduction to the bottle is overlapping (16–23 and 20–27 days from eclosion). The change in the maintenance procedure did not affect the measure of offspring production, as we continued to count the emerging flies for 28 days after the removal of the parental flies, and always measured offspring production relative to the control population (N500) that was maintained by the same procedure as the concurrent experimental populations (N10 and N40; see Offspring production).

### Offspring production

Offspring production was counted for 28 days after removing the parental flies from the bottles. Offspring production of the N10 populations was counted from all available bottles, including the extra bottles (see Population maintenance), in order to improve the accuracy of the measure. Thus, the number of bottles used for counting population offspring production was one to four for the N10 populations (most often two), four for the N40 populations, and 50 for the control population. The per capita offspring production was obtained by dividing the total number of offspring with the number of bottles in the replicate (in other words, we thus measured per bottle offspring production, but as each bottle had five pairs of parental flies, our measure corresponds to per capita offspring production). For the N10 and N40 populations, we calculated the per capita offspring production relative to the per capita offspring production of the control population (N500) measured at the same generation in order to control for possible environmental variation over time (Lynch and Walsh 1998, p. 263; Crnokrak and Barrett 2002). When a population went extinct, offspring production was recorded as zero from the extinction onwards.

### Extinction

A population was considered extinct if fewer female and/or male offspring eclosed during the 7-day collection period than required for founding the subsequent generation at the defined population size. The N10 populations were considered extinct only if none of the bottles (including the extra bottles) produced enough flies. This procedure results in lower extinction rates than would have been obtained if only one bottle would have been used, and the estimated extinction rates in the N10 populations can thus be considered conservative. The use of all available N10 bottles makes it easier to assess the role of genetic factors in affecting the extinction rates in the two population sizes. Stochastic variation in offspring production in single bottles (N10 populations) would exceed the stochastic variation in the means of four bottles (N40 populations). The use of single bottle would thus give more emphasis on environmental and demographic stochasticity in determining extinction rates in the N10 populations, instead of inbreeding depression and genetic load, which are the focus of this study.

### Estimation of N_e_ and inbreeding coefficient

As the effective population size of the study populations may deviate from the census size (*N* = 10, *N* = 40, and *N* = 500) due to nonrandom contribution of the parental flies to the next generation, we estimated effective population sizes (N_e_) and inbreeding coefficients (*f*), that is, the expected increase in homozygosity due to finite population size, from genetic variation at eight nuclear microsatellite loci. The eight loci chosen for the study (Vir4, Vir11, Vir32, Vir38, Vir99, Mon6, Mon17, Mon26) were polymorphic in the original large population (for details, see Routtu et al. 2007). The loci showed no evidence of linkage disequilibrium (tested using data from the control population at generation 1 with Fstat v.2.9.3, Goudet 2001).

We genotyped samples of individuals from all population sizes at multiple time points (see Table 1). Genomic DNA was extracted from flies preserved in 70–95% ethanol. After air-drying to remove traces of ethanol, the individuals were crushed in a microcentrifuge tube with a hand-held pestle. Qiagen DNeasy Tissue Kit reagents were used for extraction following the manufacturer's protocol modified for use with the Kingfisher magnetic particle processor (Thermo Scientific, Waltham, Massachusetts). The polymerase chain reactions (PCR) were carried out in a volume of 10.5 μL. The reaction mix contained 1× Mg-Free Buffer (Biotools, Madrid, Spain), 200 μmol/L dNTPs (Fermentas, Helsinki, Finland), 1 μmol/L R-primer, 1 μmol/L F-primer, 1.5 mmol/L MgCl_2_ (Biotools), 1 unit of Taq DNA Polymerase (Biotools), and 1 μL template DNA. The thermocycling conditions included: initial denaturation at 94°C for 3 min followed by 30 cycles of denaturation at 94°C, annealing at 52°C (Mon26) or at 55°C and extension at 72°C, and a final extension at 72°C for 10 min; using Bio-Rad thermocyclers (C1000 or S1000). The PCR products were denaturated with formamide together with GeneScan™ 500 LIZ™ Size Standard, separated using an ABI Prism 3130xl Genetic Analyser, and visualized using GeneMapper v.4.0 software (all Applied Biosystems, Carlsbad, California).

**Table 1:** The results of the genetic analysis of eight nuclear microsatellite loci

Pop. size	Gen.	*n*_replicates_	*n*_samples/replicate_ mean (min)	*n*_samples/locus_ mean (min)	H_e_ (SE)	H_o_ (SE)	*f*_observed_
N10	4	16	13.9 (10)	154.8 (54)	0.549 (.075)	0.453 (.067)	0.133
	7	16	11.6 (9)	128.1 (27)	0.507 (.078)	0.364 (.058)	0.303
	15	12	11.6 (1)	94.8 (39)	0.500 (.098)	0.195 (.051)	0.627
N40	13	11	14.5 (13)	112.9 (39)	0.505 (.081)	0.404 (.065)	0.227
	24	10	5.5 (5)	48.8 (28)	0.527 (.064)	0.320 (.050)	0.388
Control (N500)	1	1	105	92.1 (77)	0.523 (.083)	0.512 (.087)	0.021
	6	1	24	38.5 (32)	0.542 (.089)	0.562 (.084)	−0.075
	24	1	38	33.5 (17)	0.535 (.085)	0.505 (.073)	0.033

Pop. size, population size treatment; Gen., sampled generation; *n*_replicates_, number of replicate populations sampled; *n*_samples/replicate_, number of samples per replicate population (mean and minimum); *n*_samples/locus_, number of samples per locus (mean and minimum); H_e_, expected heterozygosity (replicate populations pooled); H_o_, observed heterozygosity (replicate populations pooled); *f*_observed_, inbreeding coefficient calculated as *f*_observed_ = 1–H_o_/H_e(N500,1)_, where H_e(N500,1)_ is the expected heterozygosity in the control population at generation 1.

Observed (H_o_) and expected heterozygosities (H_e_) were calculated using GenAlEx v.6.41 software (Peakall and Smouse 2006), pooling the data from the replicate populations (Table 1). We then determined the inbreeding coefficient for each genotyped generation as *f*_observed_ = 1–H_o_/H_e(N500,1)_, where H_e(N500,1)_ is the expected heterozygosity in the control population at generation 1 (Table 1). We then calculated the effective population size (N_e_) that would produce the observed inbreeding coefficient, using the equation *f*_t_ = *f*_t−1_ + (1–2 *f*_t−1_ + *f*_t−2_)/2N (Crow and Kimura 1970, p. 102), replacing N with different values of N_e_ and assuming that the parental flies at generation 0 were not related.

For the N40 populations, both values of *f*_observed_ (at generations 13 and 24) led to an estimated N_e_ of 23.2. For the N10 populations, all three values of *f*_observed_ led to different estimates of N_e_. As the first value of *f*_observed_ (at generation 4, estimated N_e_ = 10.4) was obtained after only four generations of isolation, and the last value of *f*_observed_ (at generation 15, estimated N_e_ = 6.9) was obtained with inadequate data due to extinctions and low offspring production in the replicate populations (see Table 1), we used the value of *f*_observed_ obtained at generation 7, which gave 8.1 as the N_e_ in the N10 populations. The estimated rate of inbreeding was thus 2.86 times faster in the N10 populations than in the N40 populations (ratio of the effective population sizes 23.2/8.1). The control population (N500) sustained a high level of heterozygosity throughout the experiment and conformed to Hardy–Weinberg expectations at all generations. Based on the observed inbreeding coefficient at generation 24 (*f*_observed_ = 0.033), N_e_ in the control population was approximately 342 individuals.

The inbreeding coefficients (*f*) in the N10 and N40 populations for generations 1–25 were calculated using the equation *f*_t_ = *f*_t−1_ + (1–2 *f*_t−1_ + *f*_t−2_)/2N (Crow and Kimura 1970, p. 102), replacing N with the estimated N_e_ and assuming that the parental flies at generation 0 were not related. The inbreeding coefficient in the control population (N500) was assumed to be negligible throughout the experiment.

### Statistical analyses

One of the N40 replicate populations was lost at an early stage of the experiment due to an accident during population maintenance, and was excluded from all analyses. In addition, one of the N10 populations was accidentally lost at generation 14. This population was excluded from the survival analysis (see below). Therefore, the number of replicates used was 11 for the N40 populations and 15 or 16 for the N10 populations, depending on the analysis. All analyses were performed with SPSS PASW Statistics 18.

For analyzing the effect of population size on offspring production and extinction at the same inbreeding coefficients, we chose to match the generations of the N10 and N40 populations according to the inbreeding coefficients of the offspring cohort (rather than the parental cohort). This choice was based on the expectation that the effects of deleterious recessive alleles will be most strongly manifested in the early survival of individuals (see, e.g., Ballou 1997). The generations at which the inbreeding coefficients calculated from the estimated effective population sizes (see Estimation of N_e_ and inbreeding coefficient) were as similar as possible between the two population sizes were generations 1–9 for the N10 populations, and generations 3, 6, 9, 11, 14, 17, 20, 23, and 25 for the N40 populations. The estimated inbreeding coefficients (*f*) of the offspring cohort at these generations were 0.06, 0.12, 0.17, 0.22, 0.26, 0.30, 0.34, 0.38, and 0.42 in the N10 populations, and 0.06, 0.12, 0.18, 0.21, 0.26, 0.30, 0.35, 0.39, and 0.41 in the N40 populations, respectively. In addition to the analyses based on the inbreeding coefficients calculated from the effective population sizes, we also analyzed offspring production using inbreeding coefficients calculated from population census sizes (*N* = 10 and *N* = 40) using the equation *f*_t_ = *f*_t−1_ + (1–2 *f*_t−1_ + *f*_t−2_)/2N (Crow and Kimura 1970, p. 102).

We performed repeated measures analysis of variance (ANOVA) to test the effect of population size and inbreeding coefficient on offspring production. Independent-samples *t*-test was used to test the difference in offspring production between the N10 and N40 populations at specific inbreeding coefficients. To explore whether offspring production of a population at one level of inbreeding was a good predictor of offspring production at subsequent, higher levels of inbreeding, we constructed correlation matrices for offspring production at different inbreeding coefficients using Pearson's correlation coefficient. Extinct populations were excluded from the calculation of correlation coefficients from extinction onwards.

Proportions of extinct replicate populations were compared between the N10 and N40 populations using Fisher's exact test. To test whether time to extinction in the N10 populations could be predicted from offspring production in generations 1 to 7, we used survival analysis (Cox regression). The survival analysis was done separately for each generation. The overall significance of the relationship between offspring production and time to extinction over generations 1 to 7 was assessed meta-analytically by first transforming the significance values of the test at each generation to Z-scores (standard normal deviates), and then combining these to overall Z-score and significance value (equation 4.10, assuming equal weights, in Rosenthal 1991).

## Results

### Offspring production

**Figure 1:**
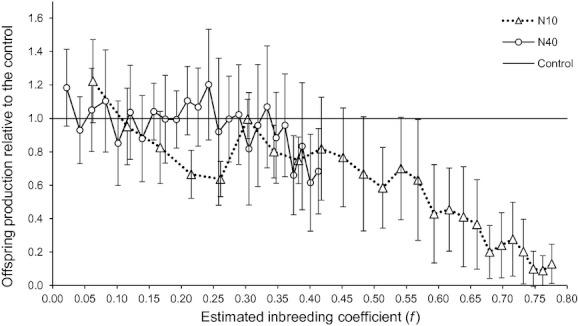
The mean per capita offspring production of the N10 and the N40 populations relative to the control population (N500) plotted against the estimated inbreeding coefficient (*f*) of the offspring generation. Error bars indicate 95% confidence intervals.

**Figure 2:**
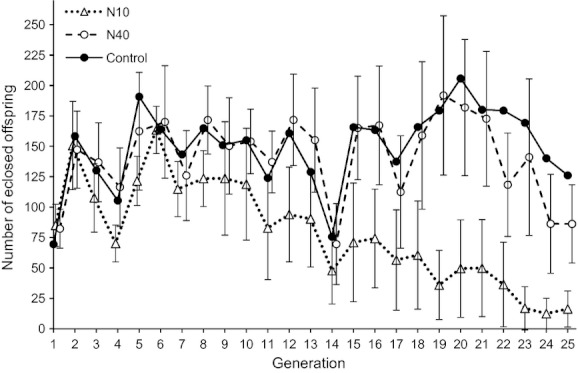
The per capita offspring production generation by generation for the N10 and N40 populations (mean with 95% confidence intervals), and for the control population (N500).

The study was continued for 26 generations, by which time the N40 populations reached *f* = 0.41, and the N10 populations reached *f* = 0.78 (all reported inbreeding coefficients are those calculated from the estimated effective population sizes). Inbreeding coefficient had an effect on offspring production, and the effect differed between the two population sizes at the inbreeding coefficients where the N10 and N40 populations could be compared (*f* = 0.06–0.42) (Table 2; Fig. 1). In the N10 populations, offspring production decreased strongly until the populations reached *f* = 0.30, when offspring production recovered to the level of the control population. The increase in offspring production between *f* = 0.26 and *f* = 0.30 was significant (Table 3). The recovery of offspring production in the N10 populations was, however, only temporary, as offspring production of the populations decreased again in later generations. In the N40 populations, no substantial decrease in offspring production was seen until after the populations reached *f* = 0.36. Independent-samples *t*-test indicated a significant difference in offspring production between the N10 and N40 populations when the estimated inbreeding coefficient (*f*) was 0.22 in the N10 and 0.21 in the N40 populations (N10 < N40; *t*_25_ = −3.939, *P* = 0.001). The results are robust to the way inbreeding coefficients are determined: When generations are matched according to inbreeding coefficients calculated from population census sizes (*N* = 10 and *N* = 40), instead of the estimated effective population sizes, the results are very similar (not shown). The control population (N500) performed well to reduce the effect of unexplained (environmental) temporal variation on offspring production in the experimental populations (see Fig. 2).

**Table 2:** The results of repeated measures ANOVA to test whether the estimated inbreeding coefficient (*f* = 0.06–0.42) affected the mean relative per capita offspring production in the N10 and N40 populations, and whether this effect was different between the two population sizes

Source	Type III sum of squares	df	Mean square	F	*P*
Inbreeding coefficient	3.035	4.679	0.649	3.512	0.006
Inbreeding coefficient × Population size	2.302	4.679	0.492	2.664	0.028
Error (inbreeding coefficient)	21.602	116.974	0.185		

The data were normally distributed (Shapiro–Wilk test for normality). Mauchly's test indicated violation of the sphericity assumption ( χ^2^_35_ = 62.979, *P* = 0.003), therefore degrees of freedom were corrected using Greenhouse–Geisser estimate of sphericity (*ε* = 0.585).

**Table 3:** The within-subjects contrasts from repeated measures ANOVA to test the difference in the mean relative per capita offspring production between each consecutive inbreeding coefficient from *f* = 0.06 to *f* = 0.42 in the N10 populations

Source		Type III sum of squares	df	Mean square	F	*P*
Inbreeding coefficient	0.06 vs. 0.12	1.188	1	1.188	6.077	0.026
	0.12 vs. 0.17	0.250	1	0.250	1.501	0.239
	0.17 vs. 0.22	0.413	1	0.413	2.494	0.135
	0.22 vs. 0.26	0.014	1	0.014	0.218	0.648
	0.26 vs. 0.30	2.059	1	2.059	41.969	0.000
	0.30 vs. 0.34	0.601	1	0.601	13.435	0.002
	0.34 vs. 0.38	0.043	1	0.043	0.721	0.409
	0.38 vs. 0.42	0.076	1	0.076	0.295	0.595

**Figure 3:**
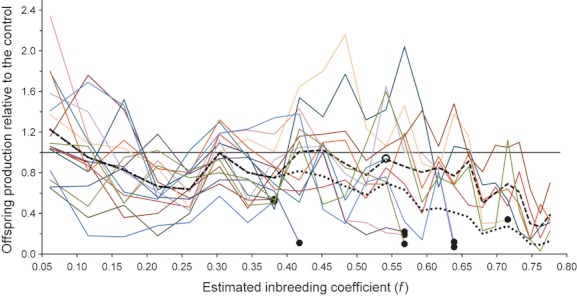
Offspring production of the N10 populations plotted against the estimated inbreeding coefficient (*f*) of the offspring generation. The solid lines of different colors represent per capita offspring production of each replicate population, the dashed black line represents the mean per capita offspring production of the persisting replicate populations when the extinct replicates are removed from the data, and the dotted black line represents the mean per capita offspring production of the replicate populations when the extinct replicates are included, but with offspring production scored as zero from extinction onwards. All values are relative to the control population (N500; marked with the horizontal solid black line). Filled circles mark the last data point before extinction of a population (note that three N10 populations went extinct at *f* = 0.42; last data point for offspring production at *f* = 0.38). Open circle marks the last data point for a replicate N10 population that was lost from the experiment due to an accident during population maintenance.

There was large variation in offspring production of the N10 populations, both among replicate populations and among generations within the replicates (Fig. 3). When extinct populations were included (with offspring production scored as zero from extinction onwards), the mean offspring production of the N10 populations decreased rather constantly after the rebound at *f* = 0.30 (Figs. 1 and 3). When extinct populations were removed from the data from extinction onwards, the mean offspring production of the persisting populations after the rebound at *f* = 0.30 was more variable, but diminished as *f* increased above 0.66 (Fig. 3).

Despite of the seemingly large variation in offspring production, for both N10 and N40 populations, the correlation coefficients between offspring production at consecutive generations were, in general, strongly positive (Tables 4 and 5, respectively; note that for the N10 populations, only the generations before the first extinctions are included). The positive correlations were especially strong and persistent over many successive generations in the N40 populations at higher inbreeding coefficients. Thus, offspring production of a population at one level of inbreeding was, in general, a good predictor of offspring production at subsequent levels of inbreeding.

**Table 4:** Pearson's correlation coefficients for per capita offspring production of the N10 populations, measured relative to the control population (N500), at estimated inbreeding coefficients from *f* = 0.06 to *f* = 0.38 (corresponding to generations 1–8; generations 9–25 were not included because of the high number of extinctions)

	*f* = 0.12	*f* = 0.17	*f* = 0.22	*f* = 0.26	*f* = 0.30	*f* = 0.34	*f* = 0.38
*f* = 0.06	.519*	.073	.309	.293	.664**	.605*	.663**
*f* = 0.12		.527*	.247	.239	.263	.386	.539*
*f* = 0.17			.328	.365	.232	.405	.159
*f* = 0.22				.452	.141	.389	.217
*f* = 0.26					.451	.411	.096
*f* = 0.30						.700**	.492
*f* = 0.34							.622*

Number of replicate populations is 16 for each value presented.

* Correlation is significant at the 0.05 level (two-tailed).

** Correlation is significant at the 0.01 level (two-tailed).

**Table 5:** Pearson's correlation coefficients for per capita offspring production of the N40 populations, measured relative to the control population (N500), at estimated inbreeding coefficients from *f* = 0.02 to *f* = 0.41 (corresponding to generations 1–25)

	*f* = 0.04	*f* = 0.06	*f* = 0.08	*f* = 0.10	*f* = 0.12	*f* = 0.14	*f* = 0.16	*f* = 0.18	*f* = 0.19	*f* = 0.21	*f* = 0.23	*f* = 0.24	*f* = 0.26	*f* = 0.27	*f* = 0.29	*f* = 0.30	*f* = 0.32	*f* = 0.33	*f* = 0.35	*f* = 0.36	*f* = 0.38	*f* = 0.39	*f* = 0.40	*f* = 0.41
*f* = 0.02	.033	.267	.486	.447	.433	.448	.189	−.202	−.279	−.227	.053	−.143	−.303	−.078	−.468	−.130	−.051	−.065	−.304	−.134	.120	.014	.260	.336
*f* = 0.04		.758**	.696*	.536	.629**	.392	.080	.090	−.073	.051	.078	.004	−.074	−.036	−.111	−.116	−.291	−.330	−.326	−.032	−.504	−.491	−.184	.235
*f* = 0.06			.784**	.681*	.462	.296	−.112	−.368	−.111	−.375	−.281	−.299	−.422	−.262	−.547	−.366	−.505	−.507	−.452	−.306	−.654*	−.682*	−.210	−.017
*f* = 0.08				.860**	.841**	.489	.306	−.015	−.240	−.071	.129	−.068	−.358	−.018	−.328	−.050	−.052	−.128	−.119	.058	−.231	−.305	.187	.389
*f* = 0.10					.849**	.701*	.610*	.131	−.021	.014	.226	.224	−.226	.211	−.166	.183	.077	.015	−.048	.125	−.158	−.198	.304	.120
*f* = 0.12						.754**	.710*	.321	−.130	.255	.435	.361	−.058	.235	.002	.218	.276	.184	.053	.339	−.025	−.047	.358	.453
*f* = 0.14							.708*	.168	.039	.109	.261	.456	.104	.115	−.057	.104	−.014	−.091	−.290	.062	−.153	−.203	.235	.005
*f* = 0.16								.612*	.102	.458	.588	.661*	.188	.495	.376	.535	.585	.513	.336	.493	.326	.344	.440	.105
*f* = 0.18									.331	.832**	.866**	.617*	.548	.770**	.866**	.817**	.670*	.656*	.607	.641*	.790**	.811**	.421	.241
*f* = 0.19										.573	.435	.651*	.824**	.603*	.577	.308	.183	.222	.348	.482	.354	.389	.339	−.018
*f* = 0.21											.941**	.772**	.819**	.712*	.845**	.653*	.645*	.754*	.571	.719*	.731*	.799**	.525	.501
*f* = 0.23												.721*	.670*	.748**	.755**	.731*	.632	.714*	.508	.639*	.763*	.787**	.588	.512
*f* = 0.24													.791**	.763**	.739**	.654*	.653*	.626	.506	.750*	.474	.547	.699*	.328
*f* = 0.26														.620*	.778**	.448	.349	.406	.362	.578	.540	.585	.445	.263
*f* = 0.27															.841**	.895**	.723*	.636*	.751*	.780**	.739*	.772**	.730*	.328
*f* = 0.29																.824**	.755*	.710*	.796**	.807**	.859**	.907**	.601	.283
*f* = 0.30																	.748*	.682*	.689*	.612	.785**	.793**	.755*	.282
*f* = 0.32																		.915**	.902**	.871**	.701*	.788**	.694*	.541
*f* = 0.33																			.776**	.726*	.676*	.813**	.564	.498
*f* = 0.35																				.882**	.730*	.785**	.631	.449
*f* = 0.36																					.618	.682*	.695*	.603
*f* = 0.38																						.964**	.619	.367
*f* = 0.39																							.575	.384
*f* = 0.40																								.615

Number of replicate populations is 11 at *f* = 0.02–0.30, and 10 at *f* = 0.32–0.41.

* Correlation is significant at the 0.05 level (two-tailed).

** Correlation is significant at the 0.01 level (two-tailed).

### Extinction

One of the 11 N40 replicate populations and three of the 16 N10 replicate populations went extinct during the time the two population sizes could be compared. The N40 population faced extinction at generation 18 (*f* = 0.32). All three N10 populations faced extinction at generation 9 (*f* = 0.42). The difference in the proportion of extinct replicate populations between the two population sizes at *f* = 0.06–0.42 was not significant (Fisher's exact test, *P* = 0.455).

Ten of the 15 N10 replicate populations went extinct by *f* = 0.78 (Fig. 3). We tested whether time to population extinction could be predicted from offspring production in generations 1 to 7 (each generation tested separately) and found that at generation 6 (*f* = 0.30), offspring production was significantly lower in populations that had less time to extinction (Table 6). This is exactly the generation at which, after a strong initial decrease, the mean offspring production of the N10 populations recovered to the level of the control population (Figs. 1 and 3). All-in-all, in six of the seven generations, offspring production was lower in populations that had less time to extinction (Table 6), and a meta-analysis of the separate tests revealed that the overall relationship between offspring production and time to extinction was significant (*Z* = 2.14, *P* = 0.032).

**Table 6:** The results of survival analysis (Cox regression) to test whether time to extinction of the N10 populations could be predicted from offspring production at generations 1–7

									95% CI for Exp(*B*)	
									
Gen.	*f* (offspring)	*n*	*B*	SE	Wald	df	*P*	Exp(*B*)	Lower	Upper
1	0.06	15	−1.103	1.002	1.213	1	0.271	0.332	0.047	2.364
2	0.12	15	−0.599	0.983	0.372	1	0.542	0.549	0.080	3.768
3	0.17	15	−0.393	0.840	0.218	1	0.640	0.675	0.130	3.506
4	0.22	15	0.026	1.415	0.021	1	0.884	1.229	0.077	19.683
5	0.26	15	−1.335	1.958	0.465	1	0.496	0.263	0.006	12.225
6	0.30	15	−4.396	1.960	5.032	1	0.025	0.012	0.000	0.574
7	0.34	15	−0.798	1.128	0.501	1	0.479	0.450	0.049	4.107

The analysis was done separately for each generation. Negative value of *B* indicates that offspring production is lower in populations that have less time to extinction.

## Discussion

Our main finding was that the increasing level of inbreeding affected offspring production differently depending on population size. In the smaller populations (*N* = 10), there was a steep decline in offspring production already at low levels of inbreeding, followed by a transient rebound to the level of the control population before a constant subsequent decline. In the larger populations (*N* = 40), offspring production decreased only after the populations reached higher levels of inbreeding.

The higher offspring production in the larger populations at low levels of inbreeding suggests that slow inbreeding is less harmful to fitness than fast inbreeding, presumably because of more effective selection in larger populations against deleterious recessive alleles (and possibly also for the maintenance of heterozygosity in overdominant loci). This finding is consistent with theoretical expectations (Falconer and Mackay 1996; Wang et al. 1999; Theodorou and Couvet 2006) and with findings of several empirical studies (Ehiobu et al. 1989; Day et al. 2003; Reed et al. 2003; Pedersen et al. 2005; Demontis et al. 2009). However, some studies have not found a clear relationship between inbreeding rate and fitness, but have found the relationship to depend on the environment and the trait studied (Bijlsma et al. 2000; Swindell and Bouzat 2006c; Mikkelsen et al. 2010; Kristensen et al. 2011). The fitness measure we used, offspring production of the whole population, combines several components of fitness and thus can be considered a relevant measure for the future survival of populations. Despite high fitness at low levels of inbreeding, offspring production did decrease at higher levels of inbreeding also in the larger populations (Fig. 1), suggesting that the larger population size did not protect the populations from the harmful effects of inbreeding and genetic drift in the long term. Unfortunately, we do not have information on viability of the larger populations at very high levels of inbreeding (*f* > 0.41).

The temporal fluctuations in the offspring production of the smaller populations (strong initial reduction followed by transient recovery and subsequent steady decline) can plausibly be explained by the combined expression and purging of highly deleterious recessive alleles during the first generations, and the accumulation and fixation of mildly deleterious mutations in later generations. Theoretical models predict that highly deleterious alleles will be effectively purged even in small populations with fast inbreeding, but purging is not effective against mildly harmful alleles (Lynch et al. 1995a; Wang et al. 1999; Theodorou and Couvet 2006). Such fluctuations of fitness in populations of limited size have not been reported often, but this may be due to the fact that only few studies have followed the fitness of small populations over a range of inbreeding coefficients. However, a similar result was recently found by Larsen et al. (2011) with guppy (*Poecilia reticulata*) populations consisting of five pairs of fish. In contrast to our finding of only a temporary recovery of fitness, the guppy populations showed a more permanent fitness recovery, suggesting that the genetic load in the fish populations consisted mainly of recessive alleles with large negative effects on fitness (Larsen et al. 2011).

Proportion of populations going extinct did not differ between the two population sizes at inbreeding coefficients where the comparison was possible (*f* = 0.06–0.42). The first extinction did not occur before *f* = 0.32 was reached, when one of the larger populations faced extinction. In the smaller populations, the first three extinctions were observed at *f* = 0.42, and after that seven more populations faced extinction within the duration of the experiment (i.e., before *f* = 0.78 was reached). Although the estimated inbreeding coefficients are average values over the replicate populations, and we do not know the exact level of inbreeding in each replicate population at the time of extinction, the results suggest that extinction risk is low at low levels of inbreeding, but increases at higher levels of inbreeding. A similar threshold relationship between extinction and the level of inbreeding has been observed in other experimental studies (see Frankham 1995), and is also expected on theoretical grounds, as the extinction risk is expected to increase sharply only after the net reproductive rate of individuals falls below one (i.e., individuals can no longer replace themselves; Lynch et al. 1995a,b; Theodorou and Couvet 2006).

In the smaller populations, there was a significant relationship between offspring production during generations 1 to 7 and time to extinction, such that lower offspring production was associated with less time to extinction. Furthermore, in both population sizes, offspring production in the replicate populations generally correlated positively over consecutive generations. These results indicate differences in genetic quality between the populations, lending credibility to the inference that differences in genetic load were contributing to among-population differences in offspring production and extinctions. The correlation coefficients of offspring production between subsequent generations were strongly positive already at low levels of inbreeding, suggesting that the differences in the genetic quality of the populations were in part due to a founder effect following the establishment of the populations. Furthermore, in the smaller populations, the relationship between offspring production and time to extinction was strongest at the generation at which the recovery in offspring production after the initial decrease occurred. This suggests that the magnitude of the fitness rebound, that is, the degree to which the population was able to purge genetic load, determined the survival of the populations in later generations. Together, the results suggest that the viability of the populations was affected by their genetic load, which in turn was influenced by stochastic founder effects, inbreeding and genetic drift, and selection.

Our results suggest that highly deleterious alleles can be purged in small populations already at low inbreeding coefficients, but that purging does not protect the small populations from eventual genetic deterioration and extinction. The results of previous studies on the effectiveness of purging in small populations are inconsistent (reviewed in Ballou 1997; Byers and Waller 1999; Crnokrak and Barrett 2002; Leberg and Firmin 2008). Although effective purging has been suggested in a number of experimental studies (Barrett and Charlesworth 1991; Latter et al. 1995; Swindell and Bouzat 2006a,b; Larsen et al. 2011), in some other studies purging has been found to be inconsistent, weak, or nonexistent. These include experimental studies on plants and animals in laboratory or semi-natural conditions (Lacy and Ballou 1998; Byers and Waller 1999; Willis 1999; Frankham et al. 2001; Radwan 2003), and studies on captive populations of animals (Ballou 1997; Boakes et al. 2007). The conflicting results may reflect differences in mutation parameters that can change between populations and traits studied, different effective population sizes used, different environmental conditions (Bijlsma et al. 1999), and the different experimental designs used to detect purging (Wang et al. 1999; Crnokrak and Barrett 2002).

The existence of natural populations that thrive in spite of severe bottlenecks in their history has also been suggested as evidence that small populations can overcome problems caused by inbreeding and genetic drift through selective elimination of deleterious alleles (Ellegren et al. 1993; Hoelzel et al. 1993; Visscher 2001; Windig et al. 2004; Facon et al. 2011). However, these populations might represent only a small fraction of bottlenecked populations, with the majority of them being extinct today. Furthermore, the success of these bottlenecked populations may rely heavily on a substantial increase in population size after the bottleneck (Bryant et al. 1990; Saccheri et al. 1996; Miller and Hedrick 2001; Reed and Bryant 2001; but see Leberg and Firmin 2008). In this study, permanent recovery of fitness was not observed in populations of consistently small size. Instead, the results show that fitness recovery in small populations was only temporary, and the populations were still at risk of extinction in later generations as inbreeding and genetic drift continued to increase the frequency and homozygosity of deleterious alleles.

The population sizes used in this study can be considered relevant for natural populations of threatened species. Due to variations in population sizes, family sizes, and sex ratio, genetically effective population sizes are often much less than population sizes of mature individuals (Frankham 2007). A rough estimate for the ratio of effective population size to estimated population size for wildlife populations is 0.2–0.3 (Mills 2007, p. 185). Hence, the smaller populations in the study, with N_e_ of 8.1, would correspond to wildlife populations with adult population size of roughly 27–40 individuals, and the larger populations with N_e_ of 23.2 to wildlife populations of roughly 77–116 individuals. According to the IUCN criteria for extinction risk (IUCN 2011), a species with a small and isolated population is considered critically endangered when population size (number of mature individuals) is estimated to be fewer than 50 individuals, and endangered when the estimated population size is fewer than 250 individuals. Our results can thus be considered representative of critically endangered and endangered species that are threatened by small population size, as far as genetic threats to population persistence are considered.

In summary, our results suggest that highly deleterious recessive alleles can be purged from small populations, and that differential purging of genetic load can predict future survival of the populations. At low levels of inbreeding, slower inbreeding was found to be less harmful for population viability, suggesting that slow inbreeding allows more effective selection against the harmful effects of inbreeding and drift. However, our results also imply that if population size remains small, inbreeding and drift will impose threats to population existence when higher levels of inbreeding are reached, regardless of the rate of inbreeding. Our continuous monitoring of the same populations with increasing inbreeding level allowed us to find this pattern, demonstrating an important methodological point that monitoring the evolution of population fitness from low to increasing levels of inbreeding, as opposed to measuring the fitness only in one point of time at a given coefficient of inbreeding, may provide a better understanding of the dynamics of genetic load in small populations.
